# Treatment trajectories by radiologic pattern of lung injury in interstitial lung disease with myositis-associated antibodies: a retrospective cohort study

**DOI:** 10.1093/rap/rkag128

**Published:** 2026-10-20

**Authors:** Kevin Grudzinski, Thomas C Bolig, Malek Shawabkeh, Kavitha C Selvan, Raymond J Goodwin, Emily Olson, Bradford C Bemiss, Nishant Parekh, Hatice Savas, Jane E Dematte, Anthony J Esposito

**Affiliations:** Division of Pulmonary and Critical Care Medicine, Department of Medicine, Northwestern University Feinberg School of Medicine; Division of Pulmonary and Critical Care Medicine, Department of Medicine, Northwestern University Feinberg School of Medicine; Division of Pulmonary and Critical Care Medicine, Department of Medicine, Northwestern University Feinberg School of Medicine; Division of Pulmonary and Critical Care Medicine, Department of Medicine, Northwestern University Feinberg School of Medicine; Division of Pulmonary and Critical Care Medicine, Department of Medicine, Northwestern University Feinberg School of Medicine; Division of Pulmonary and Critical Care Medicine, Department of Medicine, Northwestern University Feinberg School of Medicine; Division of Pulmonary and Critical Care Medicine, Department of Medicine, Northwestern University Feinberg School of Medicine; Department of Radiology, Northwestern University Feinberg School of Medicine, Chicago, IL, USA; Department of Radiology, Northwestern University Feinberg School of Medicine, Chicago, IL, USA; Division of Pulmonary and Critical Care Medicine, Department of Medicine, Northwestern University Feinberg School of Medicine; Division of Pulmonary and Critical Care Medicine, Department of Medicine, Northwestern University Feinberg School of Medicine

**Keywords:** interstitial lung disease, myositis, nonspecific interstitial pneumonia, organizing pneumonia

## Abstract

**Objectives:**

Lung involvement in interstitial lung disease (ILD) with myositis-associated antibodies (myositis-ILD) predominantly has two radiologic patterns—nonspecific interstitial pneumonia (NSIP) and organizing pneumonia (OP)—that often coexist, yet clinical outcomes of these patterns remain unclear. We aimed to describe functional and radiologic trajectories during immunomodulatory therapy by predominant radiologic pattern in myositis-ILD.

**Methods:**

In this retrospective, single-centre cohort study, we studied participants from the Northwestern University ILD Registry with a circulating myositis-associated antibody, ILD, and at least 6 months of immunomodulatory therapy during a 24-month observation period. Two thoracic radiologists assigned the predominant radiologic pattern (NSIP or OP). The primary outcome was the change in forced vital capacity (FVC); secondary outcomes were the change in diffusing capacity of the lungs for carbon monoxide (DLCO) and qualitative and quantitative (Kazerooni score) radiologic change. Groups were compared with Fisher exact and Wilcoxon rank-sum tests.

**Results:**

Forty-one participants were characterized (29 [71%] OP-predominant, 12 [29%] NSIP-predominant). Both subgroups had improved FVC on immunomodulatory therapy (OP +0.36 l [*P* = 0.006]; NSIP +0.25 l [*P* = 0.09]). DLCO did not change significantly in either subgroup (OP +1.22 [*P* = 0.11]; NSIP -0.75 [*P* = 0.37]). The OP cohort improved on qualitative imaging analysis (*P* < 0.05), driven by reduced groundglass/consolidative opacity (*P* = 0.006). Participants with NSIP/OP overlap had greater baseline radiologic severity.

**Conclusions:**

In myositis-ILD, both radiologic subgroups showed measurable functional and radiologic change during immunomodulatory therapy, with larger improvements in the OP-predominant subgroup; the magnitude of change did not differ significantly between subgroups. These descriptive, hypothesis-generating findings require multicentre validation.

Key messages

- In myositis-ILD, an OP-predominant CT pattern showed greater functional and radiologic improvement during immunomodulatory therapy.
- An NSIP-predominant pattern showed limited, non-significant functional improvement.
- These hypothesis-generating findings require multicentre validation before informing treatment decisions.

## Introduction

Interstitial lung disease (ILD) is a common complication of the idiopathic inflammatory myopathies (IIMs) associated with increased morbidity and mortality [1]. The diagnosis of myositis-associated ILD (myositis-ILD) poses a challenge fueled by the expansion of identified myositis-associated antibodies (MAAs) and recognition that ILD may precede muscle or skin involvement as the initial manifestation of IIMs [2, 3]. In participants with myositis-ILD, two computed tomography (CT) patterns of lung involvement predominate—nonspecific interstitial pneumonia (NSIP) and organizing pneumonia (OP) [3, 4]. It remains unclear whether either of these patterns is associated with clinical outcomes. This observational study describes functional and radiologic trajectories during immunomodulatory therapy in a single retrospective cohort of participants with myositis-ILD, stratified by predominant radiologic pattern (NSIP-predominant or OP-predominant). These findings characterize the natural history of these radiologic phenotypes during treatment and generate hypotheses for larger, multicentre cohorts.

## Methods

### Participant data, study criteria and assignment of radiologic pattern

Participant data were obtained from the Northwestern University (NU) ILD Registry from 01/01/2000 through 03/01/2023. The NU ILD Registry enrolled participants with confirmed ILD evaluated by the Northwestern Medicine ILD and Pulmonary Fibrosis Program who provided written informed consent for inclusion in observational research. All study procedures were approved by the NU Institutional Review Board (STU00209990).

We identified participants with ILD and circulating MAAs. The MAAs queried from the Registry were anti-PL-7, PL-12, EJ, OJ, SRP, Mi-2, U3-RNP, U2-RNP, Ku, Jo-1, TIF-1gamma, MDA5, NXP-2, PM/Scl-100, SS-A 52kDa and U1-RNP. The inclusion criteria were (1) age ≥ 18 years; (2) diagnosis of ILD; (3) an identified circulating MAA; (4) baseline pulmonary function test (PFT) and CT chest obtained at or near ILD diagnosis; (5) at least 6 months of cumulative immunomodulatory therapy during the 24-month observation period and (6) follow-up PFT and CT chest obtained at approximately 24 months. The exclusion criteria were (1) an overlap CTD or diagnosis of a separate primary CTD; (2) clinically significant emphysema (determined by consensus review of CT imaging by thoracic radiologists); (3) a pattern of usual interstitial pneumonia on CT imaging and (4) less than 6 months of follow-up on immunomodulatory therapy. Autoantibodies were assessed by the Myositis Specific 11 Antibodies Panel (Quest Diagnostics, Secaucus, NJ; Test Code: 94777). Myositis-ILD was defined operationally as ILD with a circulating MAA; a formal diagnosis of IIM by ACR/EULAR criteria was not required, and overt muscle or cutaneous involvement was not mandated for inclusion.

Two thoracic radiologists (N.P., H.S.) reviewed chest CT imaging to assess the radiologic pattern and assigned each participant to a predominant-pattern subgroup: NSIP-only or NSIP-predominant (‘NSIP-predominant subgroup’) or OP-only or OP-predominant (‘OP-predominant subgroup’). If a case had an overlap of NSIP and OP patterns, the radiologists determined which pattern predominated. Both radiologists independently reviewed the cases. In cases of disagreement, collaborative adjudication was performed by joint case review, and a consensus was reached. The radiologists were blinded to their previous assessments during adjudication. All chest CT scans were reviewed at high resolution (thin-section, ≤1.5 mm); scans not meeting this technical standard were excluded from review. Representative high-resolution CT examples of each radiologic pattern—NSIP, OP and OP-predominant and NSIP-predominant NSIP/OP overlap—are shown in Fig. 1.

**Figure 1:**
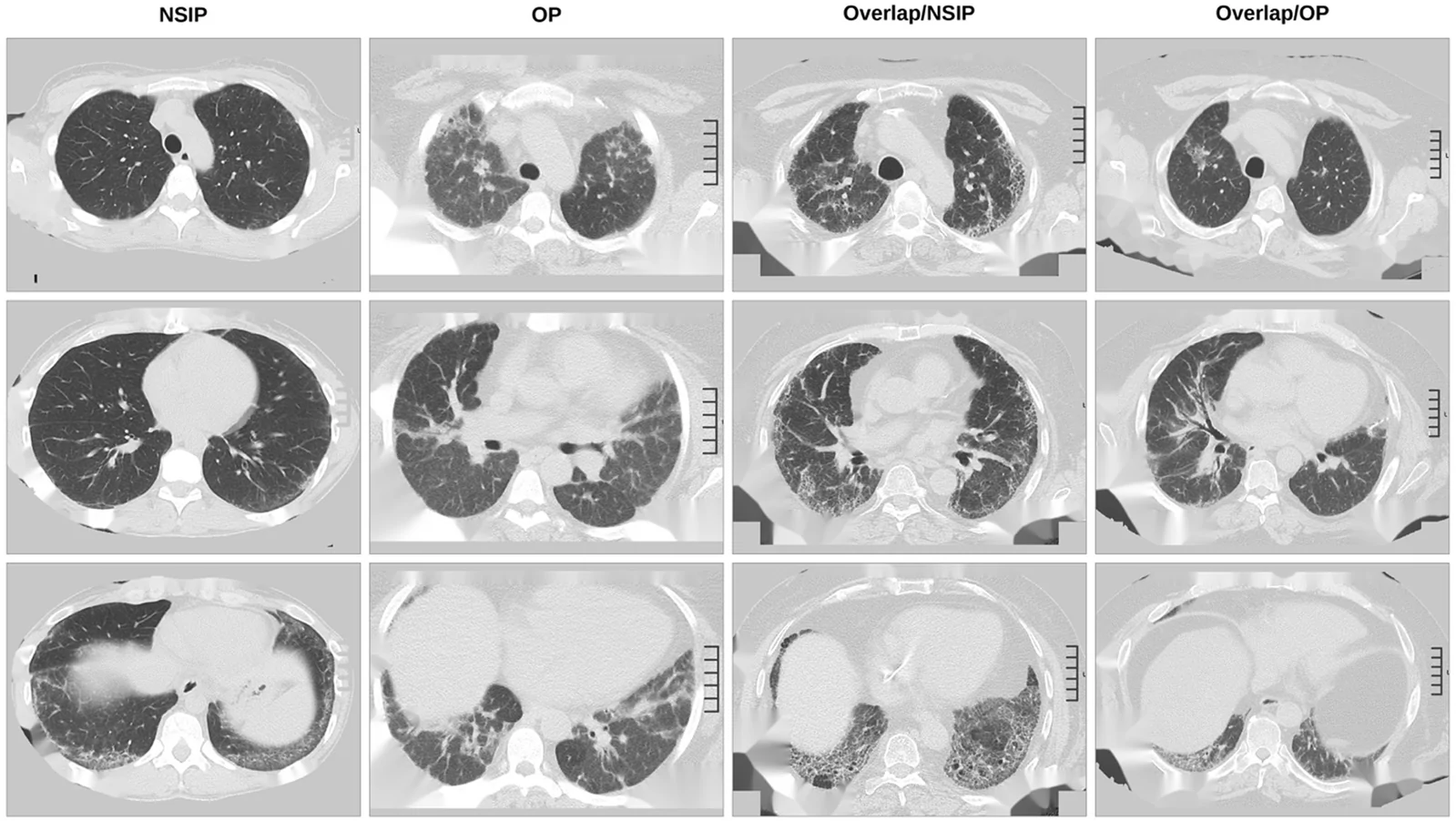
Radiologic patterns of lung injury. Representative high-resolution CT patterns of lung involvement in myositis-associated interstitial lung disease. NSIP: symmetric, basal- and peripheral-predominant groundglass opacity with fine reticulation and traction bronchiectasis, often with relative subpleural sparing. OP: patchy peribronchovascular and subpleural consolidation, frequently with air bronchograms. Overlap/NSIP-predominant: groundglass opacity and reticulation predominate, with a lesser component of consolidation. Overlap/OP-predominant consolidation predominates over a background of groundglass opacity and reticulation. Abbreviations: NSIP, nonspecific interstitial pneumonia; OP, organizing pneumonia

### Outcomes

The primary outcome was the absolute change in forced vital capacity (FVC) over 24 months. Secondary outcomes included the absolute change in diffusing capacity of the lungs for carbon monoxide (DLCO) and qualitative and quantitative radiologic progression of ILD. Radiologic progression was assessed by the thoracic radiologists via direct comparison of a participant’s CT imaging at diagnosis to a follow-up CT most adjacent to 24 months later. Progression was qualitatively assessed visually and assigned categorically as ‘progressed’, ‘stable’ or ‘improved’ on follow-up compared with the initial imaging. Quantitative assessment used the validated Kazerooni score [5], which grades the extent of groundglass and fibrotic abnormality in each lobe (0–5 per abnormality); lobar scores are summed, with higher totals indicating more extensive disease.

### Statistical analysis

Continuous variables are summarized as mean (SD) or median [interquartile range] and categorical variables as number (percent). Between-group comparisons—for the NSIP- versus OP-predominant subgroups and for participants with versus without NSIP/OP overlap—used Fisher’s exact tests for categorical variables and Wilcoxon rank-sum tests for continuous variables. Within-group changes from baseline to follow-up were assessed with the Wilcoxon signed-rank test, and between-subgroup differences in the magnitude of change (ΔFVC, ΔDLCO, and ΔKazerooni score) were compared with the Wilcoxon rank-sum test. All tests were two-tailed, with a significance threshold of α = 0.05. Analyses were performed in R (v4.4.0).

## Results

### Study population

We identified 81 participants with circulating MAA from the NU ILD Registry. Forty-one (51%) of these participants met the inclusion/exclusion criteria and were included in the analyses. The most common reason for exclusion was an overlap CTD. Participants were predominantly White, female and never smokers with an average age of 56 years (Table S1, available at *Rheumatology Advances in Practice* online). Malignancy was uncommon (5%). The most common circulating MAA was anti-Jo-1 (34%), followed by anti-PL-7 (22%), anti-EJ (12%) and anti-PL-12 (10%).

A variety of immunomodulatory agents were utilized during the follow-up period. These agents were typically used in succession (e.g. high-dose prednisone followed by a steroid-sparing agent) or in combination. All but one participant (98%) received prednisone. Most participants received tacrolimus (56%) and/or mycophenolate (51%). Other immunosuppressive agents utilized were azathioprine (44%), rituximab (27%) and cyclophosphamide (7%). Intravenous immunoglobulin was administered to 34% of participants.

Twenty-nine participants (71%) were assigned to the OP-predominant subgroup, while 12 (29%) were assigned to the NSIP-predominant subgroup. Fifteen (37%) had an NSIP/OP overlap pattern, which was further adjudicated into a predominant pattern for subgroup assignment; most of the overlap patterns were assigned to the OP-predominant subgroup (*n* = 11; 73%). Demographics, MAA distribution and treatment with immunomodulatory agents did not differ significantly between the subgroups (all *P* > 0.05; Table S1, available at *Rheumatology Advances in Practice* online). The OP-predominant subgroup had slightly shorter median time to follow-up PFT and CT.

### Primary outcome

Over the 24-month follow-up period, both subgroups had an increase in mean FVC (Fig. 2A). In the OP-predominant subgroup, the mean absolute change in FVC was +0.36 l (SD 0.58; *P* = 0.006). In the NSIP-predominant subgroup, the mean absolute change was +0.25 l (SD 0.43; *P* = 0.09). The wide SDs in both cohorts reflect substantial within-group heterogeneity in response.

**Figure 2:**
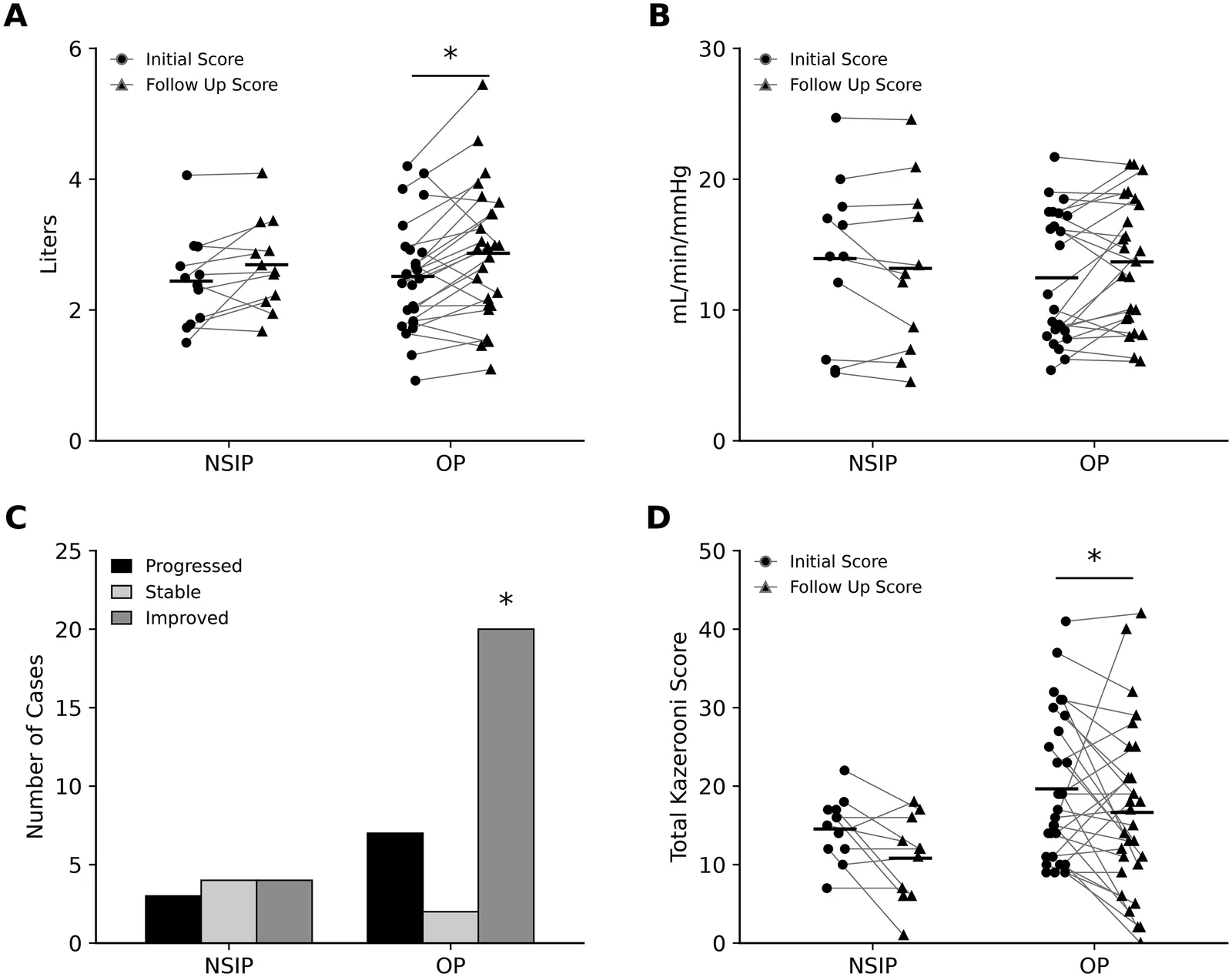
Physiologic and radiologic trajectories over 24 months. Comparisons of the NSIP- and OP-predominant cohorts by PFT and radiologic variables. Panel A displays the change in FVC for the NSIP- and OP-predominant subgroups. Both cohorts had an improvement in FVC. The OP-predominant subgroup had a statistically significant improvement in FVC. Panel B displays the change in DLCO for both groups. The NSIP cohort had a decline in DLCO, whereas the OP cohort had an improvement. Panel C displays the distribution of cases based on visual assessment by thoracic radiologists. The OP cohort had a statistically significant number of cases that were assessed as improved. Panel D displays the change in the total Kazerooni score. Both groups had a decline in total Kazerooni score, indicating an improvement in the degree of lung involvement by assessing a combination of groundglass/consolidative attenuation and fibrosis. The OP cohort had a statistically significant improvement in the total Kazerooni score. * = *P* < 0.05. — = mean value in panels A, B and D, displayed on sides of spaghetti plots. Abbreviations: DLCO, diffusing capacity of the lung for carbon monoxide; FVC, forced vital capacity; NSIP, nonspecific interstitial pneumonia; OP, organizing pneumonia

### Secondary outcomes

The OP-predominant subgroup had a mean absolute change in DLCO of +1.22 ml/min/mmHg (SD 2.9; Fig. 2B; *P* = 0.1). Conversely, the NSIP-predominant subgroup had a non-significant mean decrease of −0.75 ml/min/mmHg (SD 1.9; *P* = 0.4) over the 24-month observation period. The OP-predominant subgroup had 20 cases that demonstrated radiologic improvement (*P* < 0.05) as visually assessed by thoracic radiologists (Fig. 2C). The NSIP-predominant subgroup did not have a significant difference among the categories of ‘progressed’, ‘stable’ or ‘improved’ lung involvement on follow-up CTs.

By quantitative analysis of radiologic imaging by Kazerooni scores, the OP-predominant subgroup had an initial mean total score of 19.7 that improved to 16.7 (Fig. 2D; *P* = 0.04). The fibrosis subscore worsened from 6.1 to 8.5 (*P* = 0.02) whereas the groundglass/consolidation subscore significantly improved from 13.5 to 8.2 (*P* = 0.0002). The NSIP-predominant subgroup had an initial mean total score of 14.5 that improved to 10.8, which approached statistical significance (*P* = 0.06). The mean change in the fibrosis subscore was 8.4 to 7.7 (*P* = 0.36), and the mean change in groundglass/consolidation subscore was 6.2 to 3.1 (*P* = 0.047).

In direct between-subgroup comparisons, the magnitude of change did not differ significantly between the OP- and NSIP-predominant subgroups for FVC (*P* = 0.35), DLCO (*P* = 0.08), or total Kazerooni score (*P* = 0.96). Only the fibrosis subscore changed differently between subgroups (*P* = 0.047), increasing more in the OP-predominant subgroup.

### Exploratory analysis of NSIP-OP overlap

Given that NSIP/OP overlap has been previously associated with progression of ILD [6], we performed an exploratory analysis of participants with overlap features (*n* = 15). As expected, those with overlap had significantly greater radiologic involvement at baseline, with a higher mean total Kazerooni score compared with those without overlap (Table S2, available at *Rheumatology Advances in Practice* online; 25.0 vs. 14.5, *P* = 0.0001). Both overlap and non-overlap subgroups demonstrated improvement in FVC over follow-up, without significant changes in DLCO. Improvement in total Kazerooni score was observed in the overlap subgroup (*P* = 0.004), largely driven by a reduction in groundglass/consolidation. Rates of hospitalization and supplemental oxygen use were numerically higher in the overlap subgroup (47% vs. 31% and 27% vs. 15%, respectively), though these differences were not statistically significant.

## Discussion

In this observational cohort study, participants with ILD, circulating MAAs and an OP-predominant pattern of lung involvement showed greater improvement in lung function and radiologic signs of inflammation over 24 months than those with an NSIP-predominant pattern. To our knowledge, this is the first study to evaluate outcomes in myositis-ILD stratified by predominant radiologic pattern while incorporating both functional and quantitative imaging measures. Blinded dual-radiologist review and quantitative CT scoring strengthen internal validity, and the findings suggest that radiologic phenotyping may be an accessible approach to anticipating therapeutic response in this heterogeneous population.

Assessing prognosis in myositis-ILD remains challenging given the rarity of the disease and the paucity of large studies [3, 7]. Participants with an OP-predominant pattern had a significant increase in FVC with qualitative and quantitative radiologic improvement. Although participants with an NSIP-predominant pattern showed improvement in FVC, this change did not reach statistical significance; nevertheless, quantitative measures suggested improvement in the groundglass/consolidation component within the NSIP-predominant subgroup. It is important to highlight that these differences may reflect the baseline composition of lung involvement rather than differential treatment response: the OP-predominant cohort had a higher baseline groundglass/consolidation burden, and OP is generally more radiologically reversible than the fibrotic component that predominates in NSIP. Given the small subgroups (NSIP *n* = 12; OP *n* = 29), heterogeneous regimens, and the absence of methods to balance treatment exposures (e.g. propensity-score matching), these between-group observations are hypothesis-generating and should not be interpreted as evidence of differential therapeutic responsiveness.

Although a substantial subset of our cohort exhibited NSIP/OP overlap features, evaluation of overlap was not a primary objective. Prior CTD-ILD literature has associated overlap with worse outcomes [6, 8], and, consistent with this observation, participants with overlap had significantly greater baseline radiologic involvement and numerically higher rates of hospitalization and supplemental oxygen use, without statistically significant differences.

Several limitations constrain the inferences that can be drawn. The small subgroups limit statistical power. Although broad treatment categories were similar between groups, we did not characterize cumulative steroid dose, duration of individual agents, sequencing or combination therapy, nor did we employ propensity-score matching to balance exposures; unmeasured treatment differences may have contributed to the trajectories observed. Comorbidities that can independently affect functional trajectory—notably pulmonary hypertension and respiratory muscle weakness—were not systematically assessed. As a retrospective single-centre study, it is subject to referral and treatment selection biases. Regimens were heterogeneous, precluding attribution of response to specific MAAs or individual agents, and some participants had their first follow-up PFT after immunomodulation was initiated during an index hospitalization, potentially attenuating the observed change. Finally, the 23-year enrolment period (2000–23) spans substantial evolution in the management of CTD-ILD (including shifts towards early mycophenolate, rituximab, calcineurin inhibitors, and antifibrotics), as well as advances in high-resolution computed tomography acquisition and autoantibody assay platforms, and these temporal changes may have influenced the observed trajectories. In addition, because myositis-ILD was defined by autoantibody serology without a mandatory diagnosis of inflammatory myopathy, some participants may meet criteria for interstitial pneumonia with autoimmune features rather than a defined IIM. Nevertheless, given the rarity of myositis-ILD, our cohort is comparable in size to other studies that have informed clinical management [4, 9–11].

In conclusion, in this single-centre retrospective cohort of participants with myositis-ILD, both NSIP-predominant and OP-predominant subgroups showed measurable functional and radiologic change during immunomodulatory therapy over 24 months, with larger improvements in the OP-predominant subgroup. These descriptive, hypothesis-generating findings should not be interpreted as evidence of differential therapeutic response between radiologic patterns. Larger, multicentre studies with detailed treatment characterization are needed to determine whether radiologic pattern, antibody profile and treatment strategy can be integrated to guide management in this complex disease.

## Author contributions

Anthony J. Esposito and Jane E. Dematte conceived and designed the study. Kevin Grudzinski, Thomas C. Bolig, Malek Shawabkeh, Kavitha C. Selvan, Raymond J. Goodwin and Emily Olson contributed to data acquisition and analysis. Nishant Parekh and Hatice Savas performed the thoracic radiologic assessments. Kevin Grudzinski and Thomas C. Bolig drafted the manuscript. All authors critically revised the manuscript for important intellectual content and approved the final version.

## Supplementary Material

rkag128_Supplementary_Data

## Data Availability

The data utilized for analyses in this manuscript will be shared upon reasonable request to the corresponding author.
