# Supplementary material for: Treatment trajectories by radiologic pattern of lung injury in interstitial lung disease with myositis-associated antibodies: a retrospective cohort study
Source: Rheumatol Adv Pract. 2026 Oct 20;10(4):rkag128. doi: 10.1093/rap/rkag128 (PMC13723333; doi:10.1093/rap/rkag128)
Supplement: Supplementary file 1 [file rkag128_supplementary_data.docx]

**Supplementary Material**

*Treatment Trajectories by Radiologic Pattern of Lung Involvement in Interstitial Lung Disease with Myositis-Associated Antibodies: A Retrospective Cohort Study*

**Supplementary Table S1. Study population characteristics.**

| **Characteristic** | **Overall (N=41)** | **NSIP-predominant (N=12)** | **OP-predominant (N=29)** | **p*** |
| --- | --- | --- | --- | --- |
| Age, mean (SD), yr | 56 (15.7) | 53 (18.9) | 57 (14.3) | 0.66 |
| BMI, mean (SD) | 29.8 (6.6) | 30.8 (7.9) | 29.3 (5.7) | 0.77 |
| **Sex, n (%)** |  |  |  | **0.33** |
| Female | 22 (54) | 8 (67) | 14 (48) |  |
| Male | 19 (46) | 4 (33) | 15 (52) |  |
| **Race, n (%)** |  |  |  | **0.73** |
| Asian | 4 (10) | 1 (8) | 3 (10) |  |
| Asian-Indian | 1 (2) | 1 (8) | 0 (0) |  |
| Black | 11 (27) | 3 (25) | 8 (28) |  |
| Caucasian | 20 (49) | 5 (42) | 15 (52) |  |
| Latino | 2 (5) | 0 (0) | 2 (7) |  |
| Other | 3 (7) | 2 (17) | 1 (3) |  |
| **Smoking status, n (%)** |  |  |  | **1.00** |
| Current | 1 (2) | 0 (0) | 1 (4) |  |
| Former | 15 (37) | 3 (25) | 12 (41) |  |
| Never | 25 (61) | 9 (75) | 16 (55) |  |
| **Comorbidities, n (%)** |  |  |  |  |
| Cardiac | 8 (20) | 2 (17) | 6 (21) | 1.00 |
| CKD | 3 (7) | 1 (8) | 2 (7) | 1.00 |
| GERD | 10 (24) | 4 (33) | 6 (21) | 0.44 |
| Pulmonary (asthma, COPD, PH, OSA) | 4 (10) | 1 (8) | 3 (10) | 1.00 |
| Malignancy | 2 (5) | 2 (17) | 0 (0) | 0.08 |
| **Autoantibody, n (%)** |  |  |  |  |
| Anti-EJ | 5 (12) | 0 (0) | 5 (17) | 0.30 |
| Anti-Jo-1 | 14 (34) | 5 (42) | 9 (31) | 0.72 |
| Anti-MDA5 | 1 (2) | 0 (0) | 1 (4) | 1.00 |
| Anti-Mi-2 | 2 (5) | 1 (8) | 1 (4) | 0.51 |
| Anti-OJ | 2 (5) | 0 (0) | 2 (7) | 1.00 |
| Anti-PL-7 | 9 (22) | 2 (17) | 7 (23) | 0.70 |
| Anti-PL-12 | 4 (10) | 1 (8) | 3 (10) | 1.00 |
| Anti-SRP | 3 (8) | 2 (17) | 1 (4) | 0.20 |
| Anti-TIF | 1 (2) | 1 (8) | 0 (0) | 0.51 |
| **Immunomodulatory therapy, n (%)** |  |  |  |  |
| Azathioprine | 18 (44) | 7 (58) | 11 (40) | 0.31 |
| Cyclophosphamide | 3 (7) | 1 (8) | 2 (7) | 1.00 |
| IVIG | 14 (34) | 3 (25) | 11 (38) | 0.49 |
| Mycophenolate | 21 (51) | 5 (42) | 16 (55) | 0.51 |
| Prednisone | 40 (98) | 11 (92) | 29 (100) | 0.29 |
| Rituximab | 11 (27) | 2 (17) | 9 (31) | 0.46 |
| Tacrolimus | 23 (56) | 5 (42) | 18 (62) | 0.31 |
| Tocilizumab | 1 (2) | 0 (0) | 1 (4) | 1.00 |
| **Follow-up PFT, months, median [IQR]** | 19.9 [16.8–22.3] | 21.2 [17.6–22.9] | 18.7 [16.8–21.4] | 0.27 |
| **Follow-up CT chest, months, median [IQR]** | 17.5 [10.6–21.0] | 18.3 [14.0–23.5] | 16.0 [10.0–20.7] | 0.40 |

***p** *compares the OP-predominant versus NSIP-predominant subgroups: Wilcoxon rank-sum for continuous variables (age, BMI, follow-up times) and Fisher exact for categorical variables. Sex, race, and smoking are compared at the variable level (race, Caucasian vs non-Caucasian; smoking, ever vs never).*

**Abbreviations:** *BMI, body mass index; CKD, chronic kidney disease; COPD, chronic obstructive pulmonary disease; CT, computed tomography; GERD, gastroesophageal reflux disease; IQR, interquartile range; IVIG, intravenous immunoglobulin; NSIP, nonspecific interstitial pneumonia; OP, organizing pneumonia; OSA, obstructive sleep apnea; PFT, pulmonary function test; PH, pulmonary hypertension; SD, standard deviation.*

**Supplementary Table S2. Characteristics and outcomes of cohorts with and without NSIP/OP overlap.**

| **Characteristic** | **NSIP/OP overlap (N=15)** | **NSIP or OP only (N=26)** | **p†** |
| --- | --- | --- | --- |
| **Predominant disease pattern, n (%)** |  |  | **1.00** |
| NSIP | 4 (27) | 8 (31) |  |
| OP | 11 (73) | 18 (69) |  |
| **FVC, L, mean (SD)** |  |  |  |
| Initial | 2.5 (0.9) | 2.5 (0.8) | 0.73 |
| Follow-up | 3.0 (1.1) | 2.7 (0.8) |  |
| **DLCO, mL/min/mmHg, mean (SD)** |  |  |  |
| Initial | 12.05 (6.62) | 13.50 (4.46) | 0.42 |
| Follow-up | 13.24 (6.08) | 13.71 (4.92) |  |
| **Total Kazerooni score, mean (SD)** |  |  |  |
| Initial | 24.9 (8.5) | 14.3 (5.5) | <0.001 |
| Follow-up | 17.8 (11.1) | 13.4 (8.9) |  |
| **Hospitalizations, n (%)*** | 7 (47) | 8 (31) | 0.34 |
| **Supplemental O₂, n (%)†** | 4 (27) | 4 (15) | 0.43 |

**†p** *compares participants with NSIP/OP overlap versus NSIP-or-OP only: Wilcoxon rank-sum at baseline for continuous measures and Fisher exact for categorical measures.*

**Abbreviations:** *DLCO, diffusing capacity of the lungs for carbon monoxide; FVC, forced vital capacity; NSIP, nonspecific interstitial pneumonia; OP, organizing pneumonia; SD, standard deviation.*

**Hospitalizations were defined as the number of inpatient admissions during the follow-up period.*

*†Supplemental O₂ is the number of times participants either initiated supplemental oxygen therapy or had an increase in their baseline oxygen requirements after an outpatient assessment.*
